# Economic Evaluation of an Internet-Based Stress Management Intervention Alongside a Randomized Controlled Trial

**DOI:** 10.2196/10866

**Published:** 2019-05-15

**Authors:** Fanny Kählke, Claudia Buntrock, Filip Smit, Matthias Berking, Dirk Lehr, Elena Heber, Burkhardt Funk, Heleen Riper, David Daniel Ebert

**Affiliations:** 1 Institute for Psychology Department of Clinical Psychology and Psychotherapy Friedrich-Alexander-Universität Erlangen-Nürnberg Erlangen Germany; 2 Amsterdam Public Health Department of Clinical, Neuro- and Developmental Psychology Vrije Universiteit Amsterdam Netherlands; 3 Amsterdam Public Health Research Institute Department of Epidemiology and Biostatistics VU University Medical Centre Amsterdam Netherlands; 4 Netherlands Institute of Public Mental Health Centre of Health-Economic Evaluation Trimbos Institute Utrecht Netherlands; 5 Institute for Psychology Department of Health Psychology and Applied Biological Psychology Leuphana University Lüneburg Germany; 6 GET.ON Institute for Online Health Trainings Hamburg Germany; 7 Institute of Information Systems Leuphana University Lüneburg Germany; 8 Telepsychiatric Centre University of Southern Denmark Odense Denmark

**Keywords:** work, occupational stress, economic evaluation, internet, quality of life, clinical trials, randomized

## Abstract

**Background:**

Work-related stress is widespread among employees and associated with high costs for German society. Internet-based stress management interventions (iSMIs) are effective in reducing such stress. However, evidence for their cost-effectiveness is scant.

**Objective:**

The aim of this study was to assess the cost-effectiveness of a guided iSMI for employees.

**Methods:**

A sample of 264 employees with elevated symptoms of perceived stress (Perceived Stress Scale≥22) was assigned to either the iSMI or a waitlist control condition (WLC) with unrestricted access to treatment as usual. Participants were recruited in Germany in 2013 and followed through 2014, and data were analyzed in 2017. The iSMI consisted of 7 sessions plus 1 booster session. It was based on problem-solving therapy and emotion regulation techniques. Costs were measured from the societal perspective, including all direct and indirect medical costs. We performed a cost-effectiveness analysis and a cost-utility analysis relating costs to a symptom-free person and quality-adjusted life years (QALYs) gained, respectively. Sampling uncertainty was handled using nonparametric bootstrapping (N=5000).

**Results:**

When the society is not willing to pay anything to get an additional symptom-free person (eg, willingness-to-pay [WTP]=€0), there was a 70% probability that the intervention is more cost-effective than WLC. This probability rose to 85% and 93% when the society is willing to pay €1000 and €2000, respectively, for achieving an additional symptom-free person. The cost-utility analysis yielded a 76% probability that the intervention is more cost-effective than WLC at a conservative WTP threshold of €20,000 (US $25,800) per QALY gained.

**Conclusions:**

Offering an iSMI to stressed employees has an acceptable likelihood of being cost-effective compared with WLC.

**Trial Registration:**

German Clinical Trials Register DRKS00004749; https://www.drks.de/DRKS00004749

**International Registered Report Identifier (IRRID):**

RR2-10.1186/1471-2458-13-655

## Introduction

### Background

Up to 27% of the workforce in Europe suffers from elevated stress levels [1]. According to the effort-reward imbalance model [2] and the job demand-control model [3] situations characterized by an imbalance between high effort (eg, workload) and low reward (eg, job insecurity) or high demand and low job decision latitude lead to high levels of strain. This strain is known to be a risk factor for psychological and physiological health consequences such as sleeping problems [4], mental health problems [5], cardiovascular disease [6], and chronic pain [7]. Consequently, the resulting economic burden due to productivity losses (eg, sick leave) [8] and higher health care consumption and out-of-pocket payments is substantial [9-11]. The estimated costs of work-related stress range from US $221.13 million to US $187 billion and therefore impose a tremendous burden on society [12]. Psychological interventions can be effective in reducing stress [13], but the availability of face-to-face treatments is limited [14]. Web-based and mobile-based interventions have been proposed to overcome the limitations of traditional face-to-face interventions. Such interventions are low-threshold interventions, are available 24/7, and are associated with low costs [15].

In a recent meta-analysis, it has been shown that internet-based stress management interventions (iSMIs) are effective with an effect size of *d*=0.43 (95% CI 0.31-0.51) on perceived stress [16] and a small effect on depression and anxiety, but lack evidence regarding cost-effectiveness. Internet-based interventions are often argued to be cost-effective, yet there exists little evidence. Donker et al [17] found that internet-based interventions for common mental health disorders have a considerable probability of being more cost-effective when compared with control groups. Most health economic outcome studies evaluated internet-based interventions for alcohol consumption [18], smoking cessation [19], anxiety [20], and depression [17,21]. Hedman et al compared an iSMI with an internet-based cognitive behavior therapy (iCBT) for treatment of health anxiety, where the iSMI resulted in lower costs [22].

### Objectives

To our knowledge, there exist no cost-effectiveness and cost-utility analyses of iSMIs from the societal perspective. Thus our aim was to establish the cost-effectiveness and cost-utility of this iSMI for employees.

## Methods

### Design

This study is a health-economic evaluation with a 6-month time horizon from a societal perspective alongside a 2-arm randomized controlled trial (RCT) in Germany to establish the cost-effectiveness and cost-utility of an iSMI for employees with elevated work-related stress in combination with usual care compared with a waitlist control condition (WLC) with access to treatment as usual [23]. The present health-economic evaluation followed guidelines from the International Society for Pharmacoeconomics and Outcomes Research RCT-cost-effectiveness analysis Task Force report and the recommendations of the Consolidated Health Economic Evaluation Reporting Standard [24,25]. The trial included 264 participants who were randomly allocated in a 1:1 ratio with a block size of 2 to either iSMI or WLC. An independent researcher not otherwise involved in the study performed the randomization using randomization software (Randlist, Datinf GmbH) [26]. Participants were included in the study if they were 18 years or older, currently employed, and scored 22 or above on the Perceived Stress Scale (PSS-10). One SD (SD 6.2) above the mean (PSS-10=15.3) in a large working population [27] was chosen as a cut-off value to select participants with an elevated level of stress. The exclusion criteria were to be at risk of suicide or dissociative symptoms or having been diagnosed with a psychosis. The Ethics Committee of the Philipps-University of Marburg, Germany, approved the study. The trial was registered (DRKS00004749) in the German Clinical Trials Register.

### Intervention

The most popular models to explain work-related stress are the effort-reward imbalance and the job demand-control model. According to the effort-reward imbalance model [2], work-related stress is generated by high effort (eg, pace of work and workload) and low reward received in return (eg, inadequate salary, promotion prospects, and job security). The job demand-control model [3] identifies high demand (eg, high workload) and low job decision latitude (eg, autonomy and control over the job) as factors that lead to high levels of job strain. This strain is known to be a risk factor for adverse health consequences, such as mental health problems [5], chronic pain [7], and cardiovascular disease [6]. Ideally, job strain should be reduced by changing adverse working conditions such as small rooms and bad equipment. As changing these may be difficult, stressors on an individual level such as inadequate coping strategies can also be addressed. Interventions based on Lazarus’s transactional model aim to empower the individual to reduce or modify problems at work (ie, high effort, low rewards, or low decision latitudes). This model identifies 2 strategies of coping with stressors: problem-oriented coping, to actively change or adapt stressors, and emotion-oriented coping, to cope with negative emotions due to stressors at the workplace. Thus, the iSMI is based on 2 main components: problem solving and emotion regulation. Problem solving is an evidence-based method for dealing with such problems and has been proven to be successful in improving mental health [28]. However, employees are frequently faced with unsolvable problems, which are associated with strong negative affective reactions and require effective regulation strategies. Improvement of emotion regulation skills has been shown to be both promising for reducing psychopathological symptoms [29] and a mechanism of change in previous studies using this iSMI [30]. Deficits in emotion regulation may also be an important factor for the development and persistence of mental health symptoms [31]. Yet, emotion-focused coping is regarded as the forgotten component, whereas problem-focused coping by means of problem-solving techniques is a well-established component of most cognitive-behavioral stress management trainings.

The iSMI is based on Lazarus’s transactional model of stress and includes problem solving and emotion regulation. The intervention consists of 8 sessions composed of modules for psycho-education (session 1), problem solving (sessions 2 and 3), emotion regulation (sessions 4-6), planning for the future (session 7), and a booster session (session 8). In addition, participants could choose optional modules covering different topics, for example, time management, rumination and worrying, psychological detachment from work, and sleep hygiene. Each module takes approximately 45 to 60 min to complete. Participants were advised to complete 1 to 2 modules per week. Transfer tasks such as homework assignments were integrated into the intervention to help participants integrate learned skills into daily life. Participants received nontherapeutic feedback by an e-Coach after each completed module. E-Coaches had a degree in psychology, and feedbacks were based on a standardized manual on feedback writing. Participants could also opt in for an additional text message coach along the iSMI (eg, short relaxation exercises). A detailed description of the iSMI can be found elsewhere [32]. The clinical effectiveness of the iSMI has been positively evaluated in a series of RCTs [23,30,31,33,34].

### Outcome Measures

Self-reported measures of stress and social functioning (PSS-10 and Short-Form Six-Dimension; SF-6D) were collected at baseline (T1), post treatment (T2; 7 weeks after randomization), and 6-month follow-up (T3) using a secured Web-based assessment system (AES, 256-bit encrypted).

#### Clinical Outcome

The level of perceived stress was measured by the PSS-10 [27]. Cronbach alphas indicated that the internal consistency ranged from .70 to .91 over different measurement points in this study [30]. Symptom-free status was operationalized as scoring 2 SDs below the PSS-10 sample mean at T1 (mean 25.52, SD 3.91) [23,35].

#### Quality-Adjusted Life Years

Quality-Adjusted Life Years (QALYs) were used as the primary outcome in the cost-utility analysis. QALYs were computed using the SF-6D [36]. A QALY gain of 0.5 indicates full health throughout the 6-month trial period. The SF-6D is more sensitive to change in mild conditions than the more commonly used EQ-5D and was used for the main analysis [37].

### Resource Use and Costing

We assessed direct and indirect costs which occurred over the previous 3 months at baseline, and at 6-month follow-up. All costs were calculated in Euros for the reference year 2013 (index factor 1.04 based on the year 2010), referring to the German consumer price index [38]. Costs were converted to US dollar using the purchasing power parities reported by the Organization for Economic Cooperation and Development. For the reference year 2013, €1 was equated to US $1.29.

The Trimbos Institute and Institute of Medical Technology Questionnaire for Costs Associated with Psychiatric Illness (TiC-P) adapted to the German health care system was used [39]. This is a widely used and reliable instrument for collecting self-reported data on health care utilization and productivity losses in patients with mild to moderate mental health conditions [40-46]. The German version has been used in a number of health economic evaluations alongside randomized trials [21,41,42,44]. The standard unit cost prices were multiplied by the units of resource use for each participant. Multimedia Appendix 1 presents direct medical and direct nonmedical costs by health service type. Cumulated costs of the trial were estimated using the area under curve method to linearly interpolate 3 months costs as measured at each measurement point to cover the full follow-up period of 6 months [47].

#### Health Care Costs

Health care costs were calculated according to the guidelines of Kraut and Bock et al [48,49]. We included unit costs for a physician; a medical specialist; psychological services such as a psychiatrist and psychotherapist; and allied health services such as physiotherapy, massage, occupational therapy, as well as inpatient care and rehabilitation.

#### Medication

Unit costs of prescription drugs were calculated using the German register for pharmaceutical drugs *Rote Liste* [50]. The basis for calculating costs of prescribed medication is the pharmacy retail price accounting for a specific pharmacy and manufacture’s discount. The discount rates vary between private and statutory health insurances [48]. Therefore, we weighted the mean costs of the 3 largest packages with the same agent based on the daily defined dose by the statutory population share (88,80% of the German population are statutorily insured).

#### Intervention Costs

The provider (GET.ON Institute GmbH) of the iSMI intervention GET.ON Stress estimated the current market price of the intervention at €299 (US $386) per participant. This flat tariff covers all costs for developing and hosting the intervention plus coaching of the participants. In general, it was assumed that every participant owned a computer, had access to the internet, and used the iSMI in their leisure time after working hours. Hence, these costs were not included.

#### Patient and Family Costs

Participants self-reported the cost of their out-of-pocket expenses (eg, for over-the-counter drugs). Direct nonmedical travel costs were calculated based on self-reported data that included the used method of transportation (ie, bus, taxi, or car) and round-trip distance to reach health care services. Each kilometer by car was valued at €0.30 [51]. Time spent by participants completing the intervention and/or receiving or waiting for treatment by a physician was considered part of their leisure time. The opportunity cost of leisure time, defined as the cost associated with the next best alternative use of a particular resource, was valued at €23.10 per hour. This was based on Bock et al’s recommendations [48], which estimated these costs based on the average net wage of German employees plus their average pension and unemployment insurance contributions.

Costs incurred from a domestic help (help with daily chores) or production losses resulting from unpaid work such as informal care by friends and family were calculated using the substitution method. These costs were based on the average gross hourly wage earned by a domestic worker, as suggested by Bock et al [48]. This time was valued at €18.33 per hour.

#### Costs of Productivity Losses

Absenteeism costs were calculated by applying the human capital approach [52]. In doing so, the number of work loss days was multiplied by the participant’s average gross daily wage based on their reported monthly salary. In addition, participants reported the number of workdays for which they reported lesser efficiency. On the basis of the Osterhaus method [53], these days were multiplied by an inefficiency score, which resulted in lost-workday equivalents due to presenteeism. Subsequently, based on self-reported monthly salary, their gross wages per day were calculated and used to calculate the costs that occurred due to presenteeism.

### Statistical Analysis

This study was powered to detect a mean difference of *d*=0.35 in the primary outcome (PSS) between the groups at post measurement. Cost data are usually heavily skewed to the right, with large variance requiring very large sample sizes to test the statistical significance of cost differences. Instead, we adopted a probabilistic decision-making approach for our economic analyses [54]. This procedure takes the stochastic uncertainty of the trial data into account [55] and informs the decision makers on probabilities rather than statistical significance. Due to the 6-month follow-up period, no discounting was applied.

All analyses were conducted in accordance with the intention-to-treat (ITT) principle. Missing clinical outcome data were imputed using a Markov Chain Monte Carlo multivariate imputation algorithm with 10 estimations per missing value.

Missing cost data were imputed using the regression imputation procedure implemented in Stata to obtain the required predicted values. Predictors of outcome and dropout were identified via (logistic) regression. Differences in PSS score and symptom-free status between groups were assessed at follow-up using the Chi-square test. At baseline, mean SF-6D utility values were similar in both groups (WLC: mean 0.65, SD 0.08 and iSMI: mean 0.65, SD 0.11). Therefore, no baseline adjustments were made when calculating QALYs. Differences in QALYs between iSMI and WLC were assessed using independent samples *t* tests.

### Analysis of Cost-Effectiveness and Cost-Utility

For the cost-effectiveness analyses, the incremental cost-effectiveness ratio (ICER) was calculated as incremental costs per unit of effect (QALY and symptom-free status). Symptom-free status is meaningful for decision makers and was used as the preferred effect measure as there was no difference between beta coefficients from an OLS regression on the binary outcome compared with beta coefficients from a linear probability model in GLM (GLM: beta=.36, *P*<.001 and OLS: beta=.36, *P*<.001).

The ICER was calculated as ICER=(*costs*_iSMI_−*costs*_WLC_) /(*effects*_iSMI_−*effects*_WLC_), where costs are the cumulated *costs* over the 6-month period and *effect* are QALY gains or symptom-free status.

Stochastic uncertainty in the ICER was handled using nonparametric bootstrapping, which is a resampling technique applied to the trial data, which generates 5000 simulations of the ICER. The incremental costs and incremental effects were obtained under a bootstrapped seemingly unrelated regression equations model and allowed for correlated residuals of the cost and effect equations [56]. The 5000 bootstrap replications of costs and effects were also used to obtain 95% CIs based on the percentile method.

In a next step, the simulated ICERs were plotted in a cost-effectiveness plane. On the plane, incremental effects are depicted on the horizontal x-axis and the incremental costs on the vertical y-axis. Each dot in the cost-effectiveness plane represents 1 bootstrapped ICER.

The willingness-to-pay (WTP) threshold reflects the maximum amount the society would be willing to pay for a health benefit (eg, a symptom-free person or a QALY gained). As the WTP ceiling for gaining 1 unit of health (eg, gaining 1 QALY or obtaining symptomatic remission in 1 person) is an unknown quantity, a cost-effectiveness acceptability curve was presented, which displays the probability of the intervention being cost-effective for 1 additional unit of health gained at varying WTP ceilings. All analyses were performed using Stata version 13 [57].

#### Sensitivity Analyses

The robustness of the outcomes was assessed using several sensitivity analyses. First, we used the EQ-5D-3L (European Quality of Life 5 Dimensions 3 Level) instrument [58] for the calculation of QALYs. Second, there is uncertainty regarding the cost of the intervention due to changing demand. Therefore, we conducted sensitivity analyses assuming higher and lower interventions costs (±€100). Third, inpatient costs tend to be very high, but they were only reported by a few participants (n=9, 3.4%). Such outliers may lead to distorted outcomes results, so they were removed in the final sensitivity analysis.

## Results

### Sample

Multimedia Appendix 2 presents the baseline characteristics. Interested participants were recruited from the general working population via mass media (eg, newspaper articles and television) and with the aid of a health insurance company (BARMER) within their occupational health management program. An open-access website [59] was used to sign-up for study participation. The sample predominately consisted of full-time employed middle-aged women living with a partner. A comprehensive description of the study sample and the participant flow can be found elsewhere [23]. We did not observe any clinically relevant baseline differences between study conditions.

### Study Dropouts

The study attrition was low: 10.6% (28/264) of participants did not complete the 6-month follow-up assessment. The dropout rates between the groups, with 12.8% (17/132) in the iSMI condition and 8.33% (11/132) in the WLC condition, did not differ significantly (*χ*^2^_1_=1.4 *P*=.23).

### Outcome Measures

The iSMI improved by 9.75 (SD 6) PSS-10 stress units between pre and 6-month follow-up, whereas the WLC improved by 3.0 units (SD 6) PSS. Differences regarding symptom-free status based on the PSS-10 between groups were assessed at follow-up (iSMI: 79/132, 59.8%; WLC: 31/132, 23.5%; *χ*^2^_1_=35.9; *P*<.001; NNT (Number needed to treat) =2.75, 95% CI 2.11-3.95) [23]. However, the intervention and the WLC did not differ significantly in terms of SF-6D QALY gains (iSMI=0.35, SD 0.04 vs WLC=0.35, SD 0.35; *t*_262_=−1.625; *P*=.10).

### Costs

At baseline, mean total costs were €3239 (US $4178) in the iSMI and €3183 (US $4178) in the WLC, which is only a small difference of €56 (US $72), indicating that randomization had resulted in a well-balanced trial. Table 1 presents the average 6-month accumulated per-participant costs by study condition. The costs are clustered into health care costs, patient and family costs, and costs stemming from productivity losses. After 6 months, total incremental costs were €380 (US $490); thus, the iSMI group had less costs than WLC (iSMI: €5258 and WLC: €5642). Health care costs were, on average, higher in the iSMI group compared with WLC. Hospital admissions were a major cost driver. Regarding the patient and family costs, the iSMI had less costs than WLC. Informal care was decreased by €241 for the iSMI. Finally, productivity losses produced the highest cost differences of €487, exceeding the intervention costs, meaning that the iSMI produced less cost than WLC.

### Cost-Effectiveness

**Figure 1:**
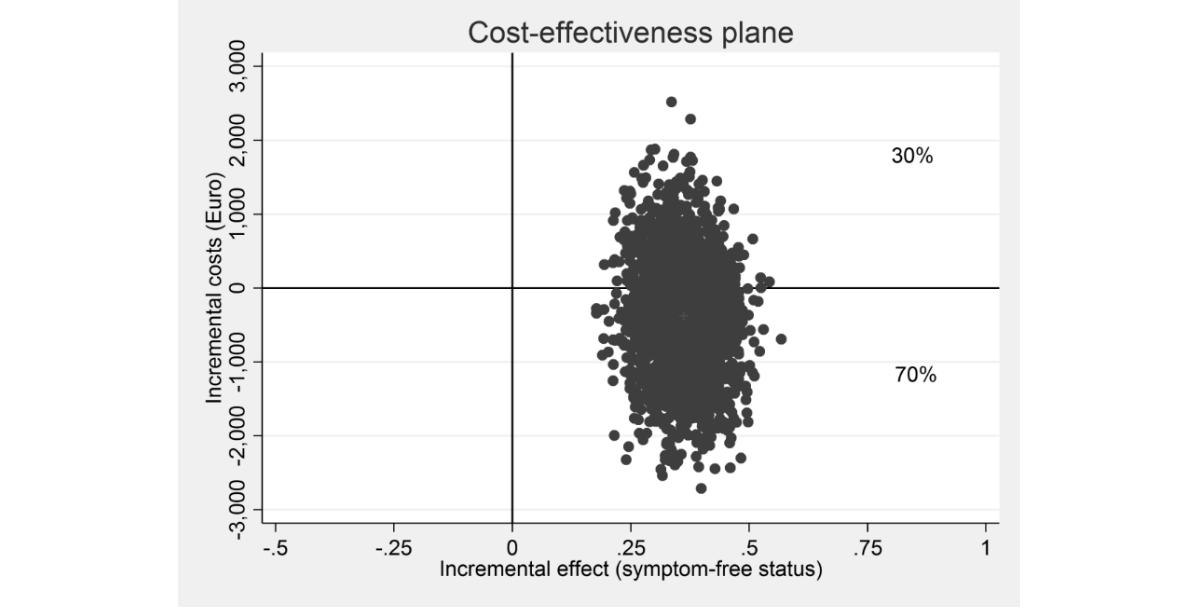
Scatterplot of 5000 replicates of the incremental cost-effectiveness ratio (mean differences in costs and symptom-free status) on the cost-effectiveness plane: internet-based stress-management intervention versus waitlist control condition.

**Figure 2:**
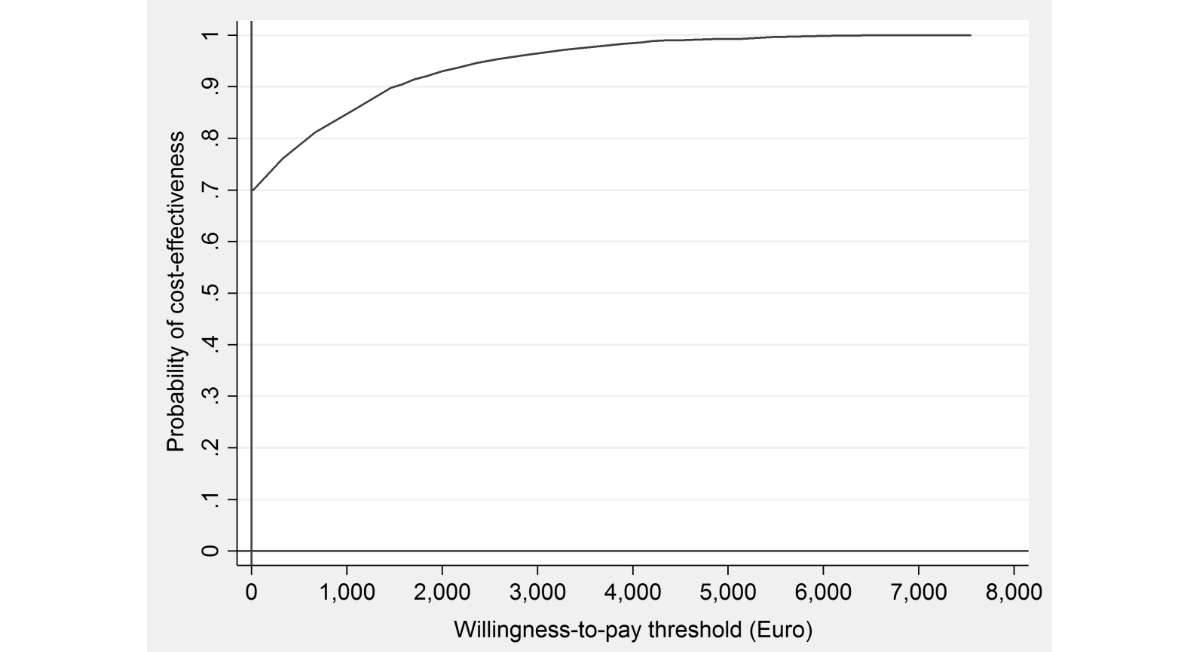
Cost-effectiveness acceptability curve showing the probability of the internet-based stress-management intervention being cost-effective at varying willingness-to-pay ceilings (based on 5000 replicates of the incremental cost-effectiveness ratio using mean differences in costs and symptom-free status).

Table 2 shows the incremental costs, effects, and cost-effectiveness ratios based on 5000 bootstrapped simulations. The bootstrapped ICER for symptom-free status on the PSS-10 was dominant. The cost-effectiveness plane is shown in Figure 1. The majority (70%) of the bootstrapped ICERs fell in the south-east quadrant, indicating a 70% probability that the intervention produces greater health at lower costs than WLC. Hence, the iSMI intervention dominates the WLC condition from a societal perspective. The remaining 30% of ICERs fell in the north-east quadrant, indicating a 30% probability that the intervention produces greater health at greater costs than WLC. Figure 2 presents the cost-effectiveness acceptability curve. If the decision maker is willing to pay €1000 and €3000 for gaining a symptom-free person, the intervention’s probability of being more cost-effective than WLC rises to 85% and 97%, respectively.

**Table 1:** Average costs per participant (in €) by condition at 6-months follow-up (area under the curve, intention-to-treat-sample, N=264).

Cost category		Internet-based stress management intervention (n=132), mean (SD)	Waitlist control condition (n=132), mean (SD)	Incremental costs, difference
**Health care costs (€)**				
	Intervention	299 (Reflects a fixed price)	0 (Reflects a fixed price)	299
	Physician services	132 (139)	147 (175)	−15
	Psychological services	111 (291)	209 (468)	−98
	Hospital in-patient	342 (2222)	188 (1237)	154
	Hospital semiresidential	234 (1444)	77 (798)	157
	Rehabilitation	8 (41)	89 (658)	−81
	Nonphysician services	167 (293)	174 (314)	−7
	Prescription drugs	50 (97)	56 (105)	−6
**Patient and family costs (€)**				
	Over the counter drugs	48 (88)	48 (78)	0
	Opportunity costs	485 (754)	526 (892)	−42
	Travel expenses	27 (48)	49 (94)	−21
	Domestic help or informal care	424 (1213)	665 (1327)	−241
**Productivity losses (€)**				
	Absenteeism	1346 (2184)	1655 (3436)	−309
	Presenteeism	1578 (1471)	1756 (1849)	−178
Total costs (€)^a^		5258 (5493)	5642 (6000)	−384

^a^Due to rounding, numbers presented may not add up precisely to the totals provided.

**Table 2:** Results of the main and sensitivity analysis based on 5000 bootstrap simulations. Costs are expressed in 2013 Euros.

Analysis and outcome		Incremental costs, € (95% CI)	Incremental effects, points (95% CI)	Incremental cost-effectiveness ratio, €/points (95% CI)^a^	Distribution over the cost-effectiveness plane, %			
					North-east quadrant^b^	South-east quadrant^c^	South-west quadrant^d^	North-west quadrant^e^
**Main analysis**								
	Perceived stress (range 0-40)	−386 (−1794 to 1006)	6.27 (4.9 to 7.7)^f^	Dominant (dominant to 171)	30	70	—^g^	—
	Symptom-free status (0/1)	−386 (−1794 to 1006)	0.362 (0.25 to 0.47)^f^	Dominant (dominant to 3360)	30	70	—	—
	QALYs^h^ (range: 0-1)	−386 (−1794 to 1006)	0.0074 (−0015 to 0.016)	Dominant^i^	26	69	2	3
**Sensitivity analysis 1^j^**								
	Perceived stress (range 0-40)	−616 (−1731 to 485)	6.27 (4.9 to 7.7^)f^	Dominant (dominant to 81)	13	87	—	—
	Symptom-free status ( 0/1)	−616 (−1731 to 485)	0.362 (0.25 to 0.47)^f^	Dominant (dominant to 1415)	13	87	—	—
	QALYs (range: 0-1)	−616 (−1731 to 485)	0.0074 (−0015 to 0.016)	Dominant^i^	12	83	2	3
**Sensitivity analysis 2^k^** **, €+100 added to intervention costs**								
	Perceived stress (range 0-40)	−286 (−1694 to 1106)	6.27 (4.9 to 7.7)^f^	Dominant (dominant to 187)	34	66	—	—
	Symptom-free status (0/1)	−286 (−1694 to 1106)	0.362 (0.25 to 0.47)^f^	Dominant (dominant to 3419)	34	66	—	—
	QALYs (range: 0-1)	−286 (−1694 to 1106)	0.0075 (−0015 to 0.016)	Dominant^i^	31	64	2	3
**Sensitivity analysis 2^k^** **, €-100 added to intervention costs**								
	Perceived stress (range 0-40)	−486 (−1894 to 906)	6.27 (4.9 to 7.7)^f^	Dominant (dominant to 155)	24	76	—	—
	Symptom-free status (0/1)	−486 (−1894 to 906)	0.362 (0.25 to 0.47)^f^	Dominant (dominant to 2764)	24	76	—	—
	QALYs (range: 0-1)	−486 (−1894 to 906)	0.0075 (−0015 to 0.016)	Dominant^i^	22	73	2	3
**Sensitivity analysis 3^l^**								
	QALYs (range: 0-1)	−386 (−1794 to 1006)	0.00186 (−0.010 to 0.014)	Dominant^i^	49	14	22	16

^a^In line with the best practice ISPOR guidelines on ‘Model Parameter Estimation and Uncertainty’ we did not report negative incremental cost-effectiveness ratios (ICERs) as they are meaningless. Instead we used the term dominant which implies that the intervention has a higher effect and less cost compared with the WLC.

^b^The north-east quadrant of the CE plane, indicating that intervention is more effective and more costly.

^c^The south-east quadrant of the CE plane, indicating that intervention is more effective and less costly.

^d^The south-west quadrant of the CE plane, indicating that intervention is less effective and less costly.

^e^The north-west quadrant of the CE plane, indicating that intervention is less effective and more costly.

^f^*P*<.05.

^g^The distribution of the ICERs (N=5000) sums to 100%. If the distribution only falls into 2 quadrants, there will not be any ICER in the other 2 quadrants (= 0%).

^h^QALYs: quality-adjusted life years.

^i^A dependably accurate 95% confidence interval for this distribution cannot be defined because there is no line through the origin that excludes alpha/2 of the distribution.

^j^Sensitivity analysis 1 analyses not including in-patient care.

^k^Sensitivity analysis 2 analyses adding €±100 of intervention costs.

^l^Sensitivity analysis 3 analyses for EQ5D quality-adjusted life years.

### Cost-Utility

**Figure 3:**
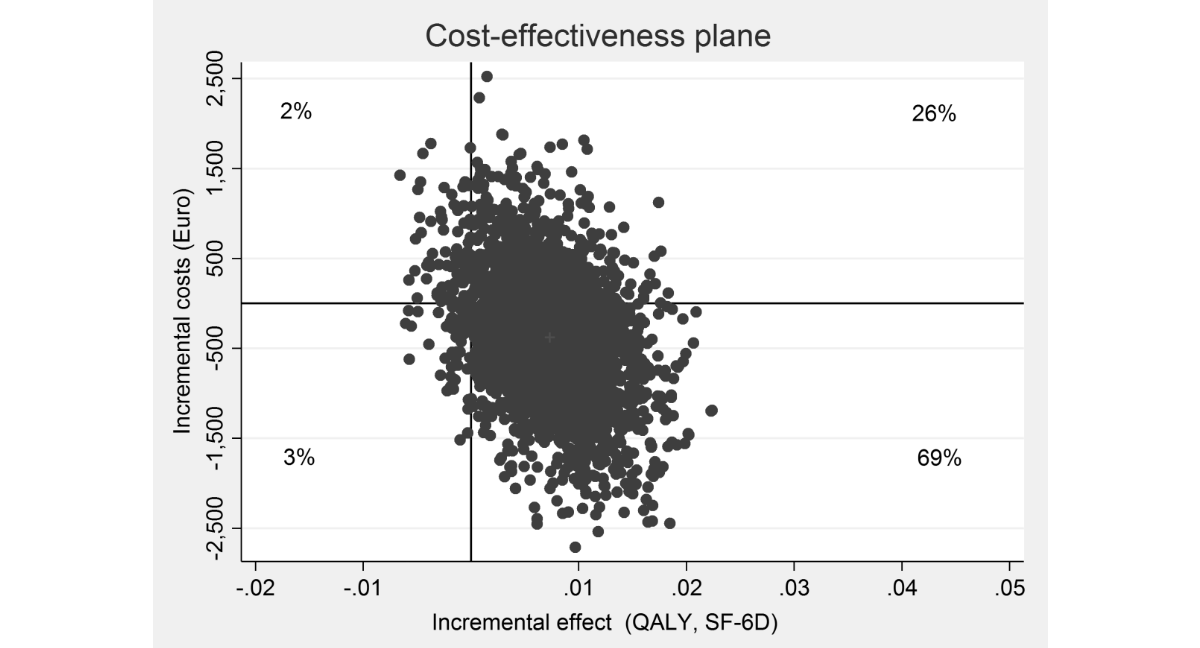
Scatterplot of 5000 replicates of the incremental cost-effectiveness ratio (mean differences in costs and quality-adjusted life years based on the Short-Form Six-Dimension) on the cost-effectiveness plane: internet-based stress-management intervention versus waitlist control condition. QALY: quality-adjusted life years; SF-6D: Short-Form Six-Dimension.

**Figure 4:**
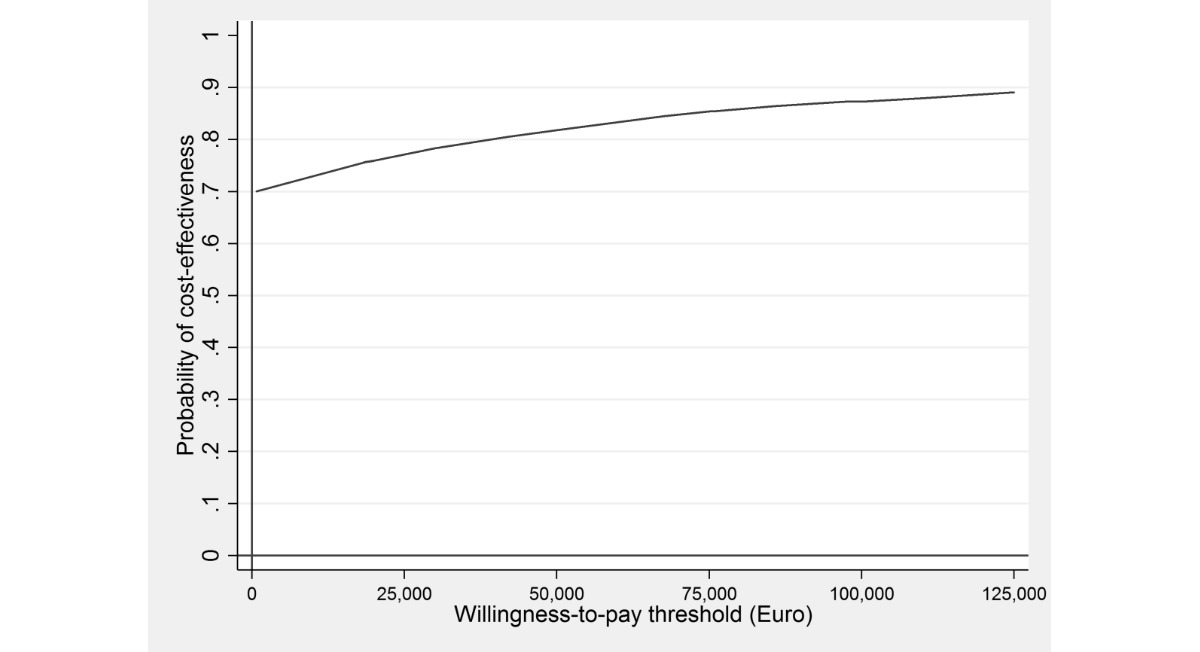
Cost-effectiveness acceptability curve showing the probability of the internet-based stress-management intervention being cost-effective at varying willingness-to-pay ceilings (based on 5000 replicates of the incremental cost-effectiveness ratio using mean differences in costs and quality-adjusted life years based on the Short-Form Six-Dimension). QALY: quality-adjusted life years; SF-6D: Short-Form Six-Dimension.

The ICER based on QALY gains showed a small health benefit (approximately 0.001 QALYs gained) for lower mean costs (€386; US $498). Of the simulated ICERs, 69% (as seen in Figure 3) fell in the south-east quadrant, reflecting the intervention’s probability of dominating WLC, whereas 26% fell in the north-east quadrant , indicating higher costs and health gains, and 2% fell in the south-west quadrant and 3% in north-west quadrant. Assuming a WTP of €10,000 and €20,000 for gaining 1 QALY, the probability rose to 73% and 76%, respectively (Figure 4).

### Sensitivity Analyses

Using the EQ-5D-3L resulted in a smaller incremental QALY gain in favor of the intervention group (0.28 QALY, SD 0.05) compared with WLC (0.28 QALY, SD 0.05), which was not statistically significant (*t*_262_=−0.296; *P*=.77). This is in line with available evidence that the EQ-5D-3L suffers from ceiling effects in milder conditions [37]. Nevertheless, at a WTP of €20,000 for gaining a QALY, the probability of being cost-effective was 71%.

As inpatient costs were reported from only a few participants but were associated with high costs, these costs might have distorted the results. Excluding these costs led to higher ICERs for both outcomes (eg, symptom-free status and QALYs). The probability of being cost-effective rose to 86% and 96% at a WTP of €0 and €1000 with regard to symptom-free status, and 86% and 90% for gaining a QALY, respectively.

Increasing and subsequently reducing the intervention costs by €100 led to a 66% and 76% probability that the intervention produces a greater health gain at lower costs than WLC with regard to symptom-free status and 1-point improvement.

## Discussion

### Principal Findings

This study evaluated the cost-effectiveness and cost-utility of a Web-based guided self-help intervention for employees with elevated stress levels aimed at reducing perceived stress compared with WLC from the societal perspective. The intervention had a significant and favorable effect on perceived stress after 6 months and a high probability of being cost-effective compared with the control condition. The overall conclusion of this study does not change when using any of the assumptions, as explored in the sensitivity analyses.

### Strengths and Limitations

First, we had missing data, which were handled using imputation techniques to perform an ITT analysis of both effects and costs [60]. As dropout rate was very low (12.8% for the iSMI and 8.33% for the WLC at 6 months), it is unlikely that this has biased the results substantially. Second, the costs and effects were only evaluated over a 6 months period. Hence, we cannot draw any conclusions about long-term effects. Third, self-reported costs and effects might have led to social desirability and/or recall bias. Nonetheless it seems unlikely that this bias differed systematically between groups due to absent baseline differences. Fourth, approaches used for cost estimation of lost productivity are based on the participants’ wages which do not reflect the average wages in the general population. Fifth, a waitlist control group design with unrestricted access to treatment as usual was chosen, which causes participants to be less motivated to initiate health-related behavior changes and thus over-accentuates effects [61]. Sixth, the majority of the sample was female, which is a common feature of mental health internet-based interventions [62]. The gender imbalance might limit the generalizability of study findings. Finally, the use of behavioral interventions does not result in improved working conditions that could cause less job strain. However, the potential of workplace-related interventions is often not fully utilized, and hence, such interventions are not systematically implemented. Thus, we recommend a combined implementation to design healthy working conditions.

### Comparison With Findings From Other Studies

The results of this study with an effect size of *d*=0.83 [23] on perceived stress are in line with the meta-analytic evidence (pooled effect size of *d*=0.43, 95% CI: 0.31-0.54) [16].

In addition, some evidence exists for the economic benefits of stress management and internet-based interventions to reduce depressive symptoms in employees. However, to the best of our knowledge, this study is the first study to evaluate the cost-effectiveness of a Web-based guided self-help intervention for employees with elevated stress levels.

Jacobsen et al evaluated the costs of a self- and professional-administered stress-management intervention not delivered over the internet in patients undergoing chemotherapy compared with usual care [63]. Lower costs and statistically higher quality of life outcomes were found in the intervention group. Hedman et al compared behavioral stress management with iCBT for treatment of severe health anxiety. The iSMI resulted in lower costs but was not considered cost-effective [22].

In a Web-based intervention by Geraedts [64], the probabilities of cost-effectiveness were 0.62 (societal perspective) and 0.55 (employer’s perspective) compared with WLC in employees with depressive symptoms. The intervention was not judged cost-effective. Besides that, the reduction of depressive symptoms was rather small (*d*=0.16) [65] compared with our study (*d*=0.64) [23] at post measurement. However, Buntrock et al reported an effect size of *d*=0.69 for a Web-based intervention for the prevention of depression. This intervention has an acceptable likelihood of being more cost-effective than enhanced usual care [21]. Focusing on perceived stress rather than on depressive symptoms in employees seems to be a cost-effective strategy to reduce the mental burden.

### Clinical Implications

The results of this study support the idea that iSMIs could be a promising cost-effective strategy in reducing adverse effects of persistent stress in the workplace. Intervention costs were mainly driven by psychologists who acted as e-Coaches. Yet, studies showed that iSMIs are also effective when delivered in a less costly adherence-focused guidance and pure self-help format [33]. However, meta-analytic evidence shows that guidance yields higher effect sizes [66]. Therefore, the cost-effectiveness of guided versus unguided iSMI needs to be evaluated.

Long-term costs caused by persistent stress, such as staff turnover or mental health disorder onsets, were not taken into account. Future studies should investigate the long-term economic effects of iSMIs. The sample consisted predominately of middle-aged women. Future research should focus on the general German working population regarding recruitment, implementation, and dissemination.

### Conclusions

This study demonstrated that this iSMI has a high probability of being cost-effective in reducing stress levels when compared with WLC. Given the increasing stress in the workplace and the small number of people who are reached via available health care services [67], it would be worthwhile to integrate such iSMIs into routine occupational health care, which conventionally only consists of face-to-face therapy by occupational health physicians.

## Appendix

Multimedia Appendix 1 Unit costs for the type of health service utilized by the participants.

Multimedia Appendix 2 Demographic characteristics: means/counts, standard deviations/percentages at baseline.

## Abbreviations

iCBT: internet-based cognitive behavior therapy
ICER: incremental cost-effectiveness ratio
iSMI: internet-based stress management interventions
ITT: intention-to-treat
PSS-10: Perceived Stress Scale
QALYs: quality-adjusted life years
RCT: randomized controlled trial
WLC: waitlist control condition
WTP: willingness-to-pay
