# Supplementary material for: Economic Evaluation of an Internet-Based Stress Management Intervention Alongside a Randomized Controlled Trial
Source: JMIR Ment Health. 2019 May 15;6(5):e10866. doi: 10.2196/10866 (PMC6707573; doi:10.2196/10866)
Supplement: Multimedia Appendix 1 [file mental_v6i4e10866_app1.pdf]

Unit costs for the type of health service utilized by the participants.

Health service type	Costs in €[a] per contact
Physician	20.57
Gynecologist	31.27
Orthopedist	25.53
Specialists for internal medicine	63.53
Ophthalmologist	36.55
Dermatologist	19.36
ETN specialist	27.80
Surgeon	44.09
Urologist	25.20
Neurologist	46.49
Psychotherapist	78.53
Dentist	54.62
Logopedics or speech therapy	40.56
Physiotherapy	17.30
Ergotherapy or occupational therapy	39.01
Mean remedies	32.29
	Costs, €[a] per day
General hospital, inpatient	648.11
Mental hospital, inpatient	348.26
General hospital, day patient	421.27
Mental hospital, day patient	226.37
Rehabilitation, outpatient	49.43
Rehabilitation, day patient	93.81
Rehabilitation, inpatient	138.19

[a] Unit costs were calculated for the year 2013 [49] or adjusted by the German consumer price index for 2013

This is a Multimedia Appendix to a full manuscript published in the J Med Internet Res for Mental Health. For full copyright and citation information see
http://dx.doi.org/10.2196/jmir.10866
