# Supplementary material for: Economic Evaluation of an Internet-Based Stress Management Intervention Alongside a Randomized Controlled Trial
Source: JMIR Ment Health. 2019 May 15;6(5):e10866. doi: 10.2196/10866 (PMC6707573; doi:10.2196/10866)
Supplement: Multimedia Appendix 2 [file mental_v6i4e10866_app2.pdf]

Demographic characteristics: means/counts, standard deviations/percentages at baseline.

	All (N=264),		Internet-based stress management intervention (n=132)		Waitlist control condition (n=132)	
	N (%)	mean (SD)	n (%)	mean (SD)	n (%)	mean (SD)
**Sociodemographic Characteristics**						
Age	—	43.3 (10.2)	—	42.4 (10.7)	—	44.2 (9.6)
Gender, female, n (%)	193 (73.1)	—	97 (73.5)	—	96 (72.7)	—
Married/Partnership	160 (60.6)	—	80 (60.6)	—	80 (60.6)	—
**Experience**						
Experience with health trainings	34 (12.9)	—	17 (12.9)	—	17 (12.9)	—
Previous psychotherapy	95 (36.0)	—	52 (39.4)	—	43 (32.6)	—
Current psychotherapy	16 (6.1)	—	5 (3.8)	—	11 (8.3)	—
**Work Characteristics**						
Years of work experience	—	18.1 (11.1)	—	17.2 (10.8)	—	18.9 (11.2)
Full—time employed	204 (77.3)	—	105 (79.5)	—	99 (75.0)	—
Part—time employed	57 (21.6)	—	25 (18.9)	—	32 (24.2)	—
On sick leave	3 (1.1)	—	2 (1.5)	—	1 (0.8)	—
**Work sectors**						
Social	97 (36.7)	—	48 (36.4)	—	49 (37.1)	—
Service	43(16.3)	—	21 (15.9)	—	22 (16.7)	—
Health	36 (13.6)	—	22 (16.7)	—	14 (10.6)	—
Economy	31 (11.7)	—	14 (10.6)	—	17 (12.9)	—
IT	15 (5.7)	—	8 (6.1)	—	7 (5.3)	—
Others	42 (16.0)	—	19 (14.3)	—	23 (17.4)	—

This is a Multimedia Appendix to a full manuscript published in the J Med Internet Res for Mental Health. For full copyright and citation information see
http://dx.doi.org/10.2196/jmir.10866
